# Evaluation of a pilot cooperative medical scheme in rural China: impact on gender patterns of health care utilization and prescription practices

**DOI:** 10.1186/1471-2458-11-50

**Published:** 2011-01-24

**Authors:** Qicheng Jiang, Zhen Jiang, Ming Zhao, Jie Tao, Chunsun Ling, Nicola Cherry

**Affiliations:** 1 School of Health Administration Anhui Medical University, Meishanlu 81, Hefei, Anhui, China; 2 Community and Occupational Medicine Program Faculty of Medicine, University of Alberta 5-30 University Terrace Edmonton, Alberta T6G 1K4, Canada

## Abstract

**Background:**

In 2003 the Chinese government introduced voluntary cooperative medical schemes (CMS), soon to be in place throughout rural China. Families who chose to enroll do so as a single unit and nothing is known about any differential effect of these new schemes on family members. This study evaluates the impact of one pilot CMS in Anhui Province on health care use by girls aged less than 5 years and women 65 years or older, and on the pattern and cost of prescriptions.

**Methods:**

Health care records were extracted covering a 10 year period, before, during and after the pilot CMS in 4 townships, one with the intervention and 3 comparison townships without. The impact of the intervention on the age and gender distribution of patients presenting for health care and on the prescription of certain drugs was assessed by logistic regression. The cost of prescriptions before, during and after the intervention period was also assessed.

**Results:**

203,058 registration and 643,588 prescription records were identified. During the intervention there was a reduced likelihood overall that a patient was female (OR = 0.92: 95%CI 0.87 - 0.97) at the intervention site. Girls aged < 5 years had an increased likelihood of health care (OR = 1.41: 95%CI 1.23 - 1.59) during the CMS, but women ≥ 65 years were relatively disadvantaged (OR = 0.84: 95%CI 0.75 - 0.95). The use of antibiotics and systemic steroids increased disproportionately at the intervention site for patients ≥ 5 years. Prescription costs at the township hospital also increased at the intervention site, particularly for older men.

**Conclusions:**

This evaluation suggests that all family members did not benefit equally from the pilot CMS and that women ≥ 65 years may be disadvantaged by the newly available reimbursements of health care costs through the CMS. It points to the need, in future evaluations, to use individuals rather than families as the unit of analysis, in order to determine whether such health care inequalities are wide-spread and persistent or are reduced in the longer term. The results also support earlier concerns about the influence of new funding resources on prescription practices and the need for regulation of for-profit prescribing.

## Background

The new cooperative medical system (CMS), first introduced by the Chinese government in 2003, had been taken up by 850 million rural residents by the end of 2008 and will be available throughout rural China by 2010 [1]. The schemes aim to prevent impoverishment by pooling funds raised from government and participants and reimbursing a proportion of health care costs[2,3]. Within national guidelines there is scope for a range of reimbursement policies: common schemes have been described in recent publications, [4-6] some of which include reports of the first evaluations [4,5,7,8]. The CMS requires families to enroll as a unit but little is known about the impact on use of health care by those of different age and gender. This may be important as, although the Chinese government works hard to raise the status of girls and women, inequalities remain: the infant mortality rate is higher for girls than boys [9] and there is less health expenditure on older women than older men [10]. A pilot CMS in western Anhui allowed us to ask whether, in this part of rural China, there was evidence of proportionately lower use of health care by young girls and older women and, if so, whether this was ameliorated by the intervention. The study also considered the impact of the pilot CMS on prescribing practices and costs.

### The intervention and study setting

The China Netherlands Poverty Alleviation Project (CNPAP) operated in one rural county in the west of Anhui Province from 1998-2003. Anhui Province has long been one of China's poorer areas, but its relative position has improved somewhat in recent years with increasing industrialization. The rural county chosen by CNPAP is geographically mixed, with flat well populated areas and irrigated agriculture in the east and mountains rising above 1,770 metres in the west. The county was first designated an official 'poverty county' in 1985, when more than half of the population was living below the official poverty line. Although economic conditions improved, more than 15% were still living in poverty in 1995. The CNPAP had the goal to devise and implement strategies to address the problems of rural poverty and environmental degradation. As part of this project a pilot CMS set up in 2000 in one township and associated villages in the mountainous west of the county, in which 75% of inhabitants were poor agricultural families. The costs of the scheme were shared between families, the project and, to a lesser extent, the county and township finance departments. Each family member paid 18 yuan in the first year and 20 yuan in the two following years; the project paid 13 yuan/head in the first year, 11 yuan in the second and 5 in the third: there was a government subsidy of 10,000 yuan in each of the three years. Some 80% of eligible families joined the scheme. The intent was to reimburse 20% of the outpatient fee from township health centres (THCs) or village clinics and 40% of THC inpatient costs (or 30% of county hospital inpatient costs), with caps of 300 yuan (outpatient) and 1000 yuan (inpatients). The project ran for 33 months from August 2000-September 2003 with a hiatus of 5 months from November 2001-March 2002. Only those living in the intervention township and associated villages could join the CMS.

Three comparison townships were identified from 14 possible sites in the same county, using information on income and size of population in 2000. As some townships had been merged in 2003 only those townships whose population could be reliably identified throughout the study period were considered as comparison sites. The three closest in size and income level to the intervention township were selected. The populations in 2000 are shown in table 1 by age and gender, together with mean per capita income. The intervention town had a population (N = 13533) very close to the mean (N= 13558) of the 3 comparison townships. The income (1289 yuan) of the intervention township was very close to the mean (weighted by population size) of the other 3 (1233 yuan). All four townships were essentially rural, with little evidence of atmospheric pollution. The age and gender distributions in the 4 townships were broadly similar, with a male excess in young children and a female excess in those age 65 years or older. At all sites health care provision was substantially underused, particularly at the township hospitals, reflecting low incomes and the need to pay 'out-of-pocket'. Residents of the 4 townships could chose to attend health centers outside their own township or, if they could afford to do so, to attend one of the county hospitals.

**Table 1:** Income, population size and female/male ratio by age group in the intervention and comparison township

	Intervention Township			Comparison Townships								
				**1**			**2**			**3**		

**Mean family income (1997)**	**1289 yuan**			**1170 yuan**			**1541 yuan**			**1118 yuan**		

**Population size (2000)**	**13533**			**12305**			**9531**			**18837**		

**Age (years)**	**N**	**Female**	**Female/Male**	**N**	**Female**	**Female/Male**	**N**	**Female**	**Female/Male**	**N**	**Female**	**Female/Male**

< 5	751	355	0.89	717	315	0.78	520	237	0.84	1248	566	0.83
5 - 14	2531	1208	0.91	2095	1021	0.95	1720	809	0.89	3890	1829	0.89
15 - 24	1489	680	0.84	932	481	1.07	795	384	0.93	1709	773	0.83
25 - 34	2724	1395	1.05	2799	1396	1.00	2100	1025	0.95	3183	1718	1.17
35 - 44	2174	1108	1.04	1947	920	0.90	1678	786	0.88	2986	1536	1.06
45 - 54	1563	781	1.00	1578	694	0.79	1160	564	0.95	2429	1179	0.94
55 - 64	1222	576	0.89	1191	505	0.74	872	403	0.86	1719	821	0.91
65 or older	1079	555	1.06	1046	547	1.10	686	346	1.02	1672	879	1.11

## Methods

The project was considered and approved by the Anhui Medical University and University of Alberta Health Research Ethics Boards (ref Pro00003937).

The study compared outcomes (gender of patient, drugs prescribed and their cost) in the intervention and comparison sites during 3 time periods: before (January 1997-July 2000) during (August 2000-September 2003) and after the intervention (October 2003-December 2006).

Registration records (one for each health care visit) for the period January 1997-December 2006 were identified at the 3 county level hospitals, 4 THC and associated village clinics. Only records for patients resident in that township were retained. Prescription records were extracted at the village and township levels but those relating to the townships of interest could not be identified at the county hospitals.

Although all available records were extracted from each level of institution (county, township, and village) throughout the 10 years, there were significant gaps in the records available. Most seriously, in the period between setting up the study and the extraction of registration records, severe flooding in the intervention township had destroyed all inpatient records and gynecological outpatient records at the THC for the periods before and during the intervention and for part of the post-intervention period (to the fall of 2005). Registration records were not available for less systematic reasons at other institutions: they simply could not be found for certain periods, particularly in the early years or, in some village clinics, had never been kept. Missing records should not (except for gynaecology) have been biased by age or gender.

For each record (registration or prescription) date, age and gender were extracted. Reason for the patient visit (diagnosis/symptom) and treatment (present for outpatients only) were extracted from registration record and cost from the prescription record. All records were extracted to worksheets and subsequently coded by post-graduate students and young faculty, assisted by junior doctors.

Diagnoses and symptoms were coded to ICD10 chapter headings or sub-classification of symptoms and signs. Treatment coding concentrated on identifying accurately drugs that were antibiotics, anti-virals and steroids for systemic use (oral or intravenous): comprehensive lists of Chinese drug names in each category were complied to help the coders.

### Statistical methods

We considered all registrations, regardless of cause, and also registrations other than those with diagnoses specific to gender; these latter included all consultations/admissions at a gynecology/obstetrics service together with all registrations coded A131 (female reproductive or breast disease), A14 (pregnancy or childbirth) or A132 (male reproductive system disease). The ratio of female/male patients was corrected for the female/male ratios in that age group within township and the resulting ratios displayed graphically, following an approach used in Manitoba [11]. The effect of the intervention was evaluated by logistic regression, estimating the likelihood of a patient being female, having allowed for time (before, during and after the intervention), township, level of institution, service (inpatient or outpatient) and age group. An interaction term indicated whether the registration was at the intervention township during the intervention period. The intervention effect specific to age was examined by adding similar interaction terms for each age group. Because the records for gynecology and obstetric clinics at the intervention site had been destroyed by flooding, the main analysis was limited to those without diagnoses specific to gender. Prescription data for in-patients at the intervention THC were not destroyed and were used to explore the robustness of conclusions drawn.

Further logistic regression models tested the effect of the intervention on the prescription of three classes of medication (antibiotics, antivirals and systemic steroids) allowing for diagnosis. The effect of the intervention on the cost of prescriptions was examined by linear regression; the cost data were skewed and the analyses repeated after natural log transformation.

The data were essentially hierarchical, with registrations and prescriptions clustered within township and institution. The full models allowing for this structure failed to converge but simplified models, using SAS, indicated only minor effects of clustering on estimates of standard error and the results presented here were from an SPSS 17.0 analysis without multilevel modeling.

## Results

The data available for analysis is given by period and township in additional file 1. The generally much larger number of prescription than registration records reflects the greater importance given to the preservation of these documents, which recorded a monetary exchange and could, at least potentially, be audited. It also reflects the inability of the data extractors to eliminate non-residents. Among the registration records of the THC at one comparison site, for example, 60% of patients were found not to be living in the township, and it must be assumed that this was the case for many of the prescriptions, including those from the intervention site.

**Figure 1:**
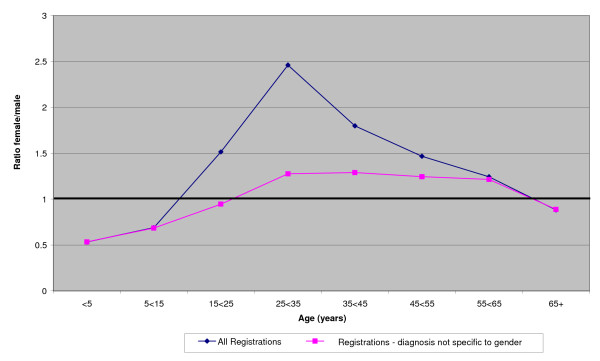
**Female/male ratios by age group for registrations in comparisons townships**: (ratios corrected for population ratios - see text)

**Figure 2:**
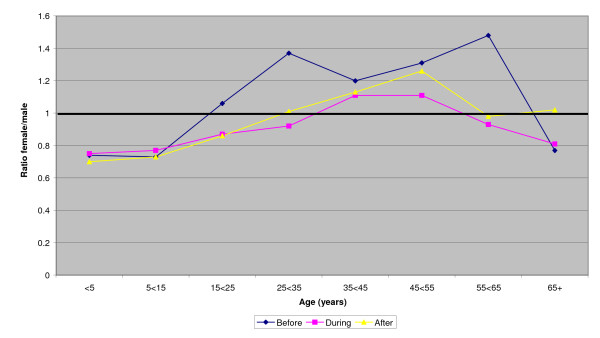
**Female/male ratios by age groups for registrations before, during and after the intervention**: intervention township. Gender determined disease and THC inpatients omitted (ratios corrected for population ratios - see text)

**Figure 3:**
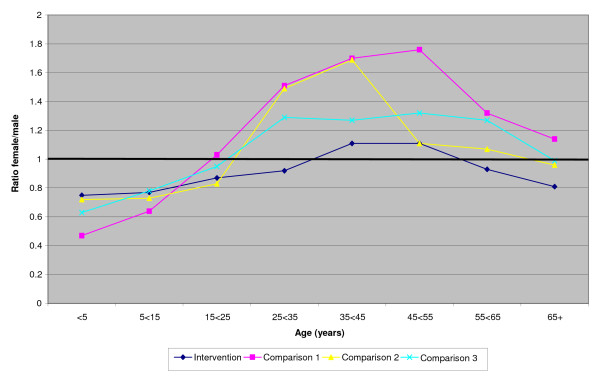
**Female/male ratios by age group for registrations during the intervention period in each Township**: gender determined disease and THC inpatients omitted (ratios corrected for population ratios - see text)

The ratio of female/male patients in the 3 comparison townships (taken together) throughout the 10 years is shown in Figure 1 both for all registrations and for those without diagnoses specific to gender. Figure 2 shows similar ratios for the intervention township, before, during and after the intervention. Figure 3 gives the ratios during the intervention period for each of the townships. In all these figures correction is made for the gender ratios by age group of the underlying populations (shown in table 1). Overall there is a clear pattern (figure 1) of health care registrations being lower for females than males in those under 15 years of age and those 65 years or older and higher for women than men in the intervening years. Removal of diagnoses specific to gender reduced the female/male ratio but it remained greater than unity in the middle years. In the intervention township the female/male ratio was reduced during the intervention period (figure 2) and was lower than in any of the comparison townships (figure 3).

Visual inspection of the data by period and institution (but without accounting for age) did not indicate a consistent female deficit (see additional file 2) but this was tested more formally in an analysis in which the outcome was that the patient was female (rather than male) and an interaction term reflected living in the intervention township during the intervention period (table 2). In the first model the interaction OR was 0.94, implying that the likelihood of a patient being female was less than expected in the intervention township during the intervention period. Model 2 included also the age of the patients, with a somewhat greater female deficit (OR = 0.92). The third model considered the interaction with age also and suggested that only young girls (< 5 years) benefited from the intervention, relative to boys of the same age (OR = 1.41). The relative disadvantage to women during the intervention period was not confined to the oldest women (OR = 0.84) but all females of 15 years or greater (as suggested by figures 2 and 3).

**Table 2:** Likelihood of a patient being female: logistic regression analysis of registration data.

	Model 1		Model 2		Model 3	
**Inpatient**	**OR**	**95% CI**	**OR**	**95% CI**	**OR**	**95% CI**

No	1	-	1	-	1	-
Yes	0.55	0.49 - 0.62	0.65	0.57 - 0.74	0.65	0.57 - 0.73
**Level**						
County	1	-	1	-	1	-
Township	1.06	0.96 - 1.16	1.20	1.09 - 1.33	1.21	1.10 - 1.33
Village	0.96	0.87 - 1.06	1.07	0.97 - 1.18	1.07	0.97 - 1.18
**Period**						
Before	1	-	1	-	1	-
During	1.07	1.04 - 1.09	1.04	1.01 - 1.06	1.03	1.01 - 1.06
After	1.03	1.01 - 1.06	1.01	0.98 - 1.03	1.00	0.98 - 1.03
**Town**						
Intervention	1	-	1	-	1	-
Comparison 1	1.01	0.97 - 1.05	1.02	0.98 - 1.06	1.02	0.98 - 1.06
Comparison 2	1.05	1.01 - 1.09	1.04	1.00 - 1.08	1.03	0.99 - 1.07
Comparison 3	0.99	0.95 - 1.03	1.02	0.98 - 1.06	1.02	0.98 - 1.06
**Interaction**						
Other	1	-	1	-	-	
Intervention site * intervention period	0.94	0.89 - 0.99	0.92	0.87 - 0.97	-	
**Age (years)**						
< 5	-	-	1	-	1	-
5 < 15	-	-	1.33	1.29 - 1.38	0.57	0.49 - 0.65
15 < 25	-	-	1.78	1.71 - 1.87	0.76	0.66 - 0.88
25 < 35	-	-	2.73	2.62 - 2.84	1.04	0.90 - 1.20
35 < 45	-	-	2.64	2.53 - 2.74	1.61	1.39 - 1.86
45 < 55	-	-	2.43	2.33 - 2.53	1.53	1.32 - 1.77
55 < 65	-	-	2.08	1.99 - 2.17	1.41	1.22 - 1.63
65 or greater	-	-	2.06	1.97 - 2.15	1.22	1.05 - 1.41
"Adult"	-	-	2.78	2.68 - 2.88	1.20	1.04 - 1.39
Unknown	-	-	1.89	1.67 - 2.14	1.61	1.39 - 1.86
Age specific intervention (site*period *age)						
< 5	-	-	-	-	1.41	1.25 - 1.59
5 < 15	-	-	-	-	1.06	0.96 - 1.18
15 < 25	-	-	-	-	0.84	0.73 - 0.97
25 < 35	-	-	-	-	0.72	0.64 - 0.81
35 < 45	-	-	-	-	0.89	0.79 - 0.99
45 < 55	-	-	-	-	0.92	0.82 - 1.03
55 < 65	-	-	-	-	0.79	0.69 - 0.90
65 or greater	-	-	-	-	0.84	0.74 - 0.95
"Adult"	-	-	-	-	0.67	0.55 - 0.82
Unknown	-	-	-	-	1.31	0.99 - 1.75

Diagnosis specific to gender & THC inpatients excluded.

The analysis was repeated including all registrations at all institutions, with age and gender from prescription records as a proxy for the destroyed registration records at the THC at the intervention site. The size of the interaction terms were essentially unchanged (model 1: OR = 0.93; 95%CI 0.90 - 0.96: Model 2 OR = 0.91; 95%CI0.88 - 0.94: Model 3 < 5years OR = 1.27; 95%CI 1.17 - 1.38: ≥ 65years OR = 0.81 95%CI = 0.76 - 0.87).

Treatment was not available for in-patients or for out-patients at the county hospital. Among outpatients at the township hospitals or village clinics, a medical treatment was recorded for more than 80% of cases at the intervention site and at 2 of the 3 comparison sites. At the first comparison site a medical treatment was recorded for only 8.4% of cases and the site was excluded from this analysis.

The most common medical treatments were patent traditional Chinese medicine (56.4% of patients) Western medicine other than antibiotics, antivirals or steroids (54.2%), antibiotics (46.2%), traditional dispensed Chinese medicine (18.4%) antivirals (10.2%) and systemic steroids (9.1%). The diagnoses most frequently recorded for these outpatients were respiratory disease (26.5%) digestive system disease (9.3%) skin disease (6.3%) injury (4.0%) and female breast and reproductive disease (3.3% overall, 6.6% in women). Symptoms (rather than diagnoses) most commonly recorded were 'general symptoms/signs' (17.5%) 'digestive and abdomen' (10.2%) and 'cardiac and respiratory' (9.5%).

The use of antibiotics, antivirals and steroids increased markedly between the first and second periods and sustained the higher level in the third. For antibiotics the rates were 36.5% (period 1), 48.6% (period 2) and 50.0% (period 3), for anti-virals 6.0%, 11.9% and 11.3% and for systemic steroids 5.8%, 10.3% and 10.1%. All 3 were prescribed more by village clinics than THCs (table 3). Antibiotics were most commonly prescribed for those aged less than 5 years and for respiratory disease, cardiac/respiratory symptoms, after injury and for digestive disease. Those living in the intervention township during the intervention period were more likely to be prescribed antibiotics (OR = 1.74) and this was maintained post-intervention (OR = 1.80) (table 3 column 1). In the intervention township antibiotics were prescribed for 60.1% of patients during the intervention and for 61.0% post-intervention. Antivirals were most commonly prescribed for respiratory and skin diseases and for those under the age of 5 years, but there was no increase in use of antivirals specifically attributable to the intervention (table 3 column 2). Systemic steroids were prescribed most frequently for those with respiratory or skin diagnoses or for patients with 'general symptoms/signs'. They were used least in patients aged < 5 years. Use was increased at the intervention township both during the intervention (OR = 1.96) and post-intervention (OR = 2.23). Use in this township was 12.5% during the intervention and 13.5% afterwards.

**Table 3:** Factors associated with the prescription of antibiotics, antivirals and systemic steroids.

	Antibiotics		Antivirals		Steroids	
Factor	**OR**	**95% CI**	**OR**	**95% CI**	**OR**	**95% CI**

**Level**						
Village	1	-	1		1	-
Township health centre	0.74	0.71 - 0.77	0.62	0.58 - 0.66	0.54	0.50 - 0.58
**Period**						
Before	1	-	1	-	1	-
During	1.07	1.02 - 1.12	1.50	1.36 - 1.65	1.30	1.19 - 1.41
After	1.11	1.06 - 1.16	1.87	1.71 - 2.05	1.16	1.07 - 1.26
**Township**						
Intervention	1	-	1		1	-
Comparison2	0.84	0.77 - 0.91	0.37	0.33 - 0.43	1.94	1.64 - 2.30
Comparison3	1.90	1.74 - 2.08	0.82	0.71 - 0.93	2.02	1.70 - 2.40
**Interaction-during**						
Other	1	-	1	-	1	-
Interventionsite*interventionperiod	1.74	1.58 - 1.92	0.74	0.64 - 0.87	1.96	1.64 - 2.35
**Interaction-post**						
Other	1	-	1	-	1	-
Interventionsite*post-interventionperiod	1.80	1.63 - 1.98	0.38	0.33 - 0.44	2.23	1.87 - 2.67
**Diagnosis/Symptom**						
A11(resp)	4.12	3.96 - 4.30	5.74	5.37 - 6.13	3.38	3.17 - 3.60
A12(digest)	2.48	2.36 - 2.62	1.06	0.94 - 1.18	1.21	1.09 - 1.34
A15(skin)	1.25	1.18 - 1.34	2.02	1.80 - 2.27	3.43	3.12 - 3.77
A131(femrepro)	0.88	0.80 - 0.96	0.20	0.13 - 0.32	0.24	0.17 - 0.34
A19(injury)	2.87	2.66 - 3.09	0.70	0.57 - 0.85	1.45	1.26 - 1.67
B8(general)	1.03	0.99 - 1.0	1.50	1.39 - 1.61	3.03	2.84 - 3.23
B2(digest & abd)	0.88	0.83 - 0.92	0.46	0.40 - 0.53	0.50	0.44 - 0.57
B1(resp & cardio)	2.66	2.53 - 2.80	1.37	1.24 - 1.51	1.12	1.02 - 1.23
**Gender**						
Male	1	-	1	-	1	
Female	0.94	0.91 - 0.97	0.98	0.98 - 1.02	0.96	0.92 - 1.01
**Age(years)**						
> 5	1	-	1	-	1	-
5 > 14	0.98	0.93 - 1.04	0.80	0.75 - 0.86	1.19	1.09 - 1.29
5 > 24	0.84	0.79 - 0.90	0.62	0.56 - 0.68	1.45	1.03 - 1.28
25 > 34	0.68	0.64 - 0.72	0.40	0.36 - 0.45	1.15	1.06 - 1.28
35 > 44	0.64	0.61 - 0.68	0.39	0.35 - 0.43	1.12	1.01 - 1.24
45 > 54	0.54	0.51 - 0.58	0.40	0.37 - 0.45	1.20	1.08 - 1.32
55 > 64	0.49	0.46 - 0.53	0.42	0.38 - 0.46	1.24	1.11 - 1.37
65+	0.46	0.43 - 0.49	0.32	0.29 - 0.36	1.19	1.08 - 1.32
"adult"	0.52	0.46 - 0.59	0.36	0.30 - 0.44	2.60	2.26 - 3.07
unknown	0.72	0.61 - 0.83	0.47	0.38 - 0.59	0.87	0.68 - 1.14

Village clinics or outpatients at a THC: those with medical treatments only.

Only 2.3% (2071/89877) of patients with a medical prescription were prescribed all three (antibiotics, antivirals and steroids), but during the intervention period this combination was prescribed for 6.0% (584/9686) at the intervention township. In a model including the factors in table 3, the OR for the interaction was 2.04 (95%CI 1.50 - 2.77). This excess was largely confined to the intervention period; the post-intervention OR was 1.21 (95%CI 0.88 - 1.65). The increased use of antibiotics and steroids in the intervention township during the intervention was very similar for males and females and occurred in all age groups except that, for steroids, there was no increase, for either boys or girls, in those aged less than five years (data not shown).

The third outcome considered was the cost of prescriptions, available for village clinics in all townships and for 3 of the 4 THCs. The median cost for western medicine was 20.0 yuan (range 0.10 - 1973) in a THC and 19.5 yuan (range 0.10 - 1385) at a village clinic. The cost of traditional Chinese medicine was higher, 32.3 yuan (range 0.20 - 1113.5) at a THC and 28.3 yuan (range 0.50 - 595.60) at a village clinic. Table 4 shows the analysis of untransformed data, where the coefficients can be interpreted as yuan. Costs increased by study period with an additional cost attributable to the intervention that was sustained post-intervention. Stratification by institution showed that this was limited to the THC. Models fitted to the natural logarithm of the costs, to allow for the right-hand-skew, supported this conclusion. Median prescription costs for the intervention site increased in the THC from 19.4 yuan (period 1) to 30.0 yuan (intervention) and 45.7 yuan (post-intervention). In the two comparison THCs the costs were 26.1 and 15.8 yuan (period 2) and 27.3 and 18.0 yuan (period 3). The increase in costs at the THC in the intervention site was much more evident for older men than women. In those aged 65 years or older the median cost for men in the intervention period was 58.15 yuan and for women 33.8 yuan. For children under 5 years the median costs in the same period were very similar (boys 20.0 yuan; girls 19.25 yuan).

**Table 4:** Factors associated with the cost of prescriptions

	All		THC		Village Clinics	
**Level**	**β**	**95% C.I**.	**β**	**95% C.I**.	**β**	**95% C.I**.

Village Clinic	-	-	-	-	-	-
THC	8.70	8.45 - 8.93	-	-	-	-
**Period**						
Before	-	-	-	-	-	-
During	6.93	6.58 - 7.28	4.40	3.93 - 4.86	3.68	3.17 - 4.19
After	14.09	13.78 - 14.41	11.48	11.05 - 11.91	11.16	10.70 - 11.62
**Township**						
Intervention	-	-	-	-	-	-
Comparison 1	-1.37	-2.11 - -0.62	-	-	2.19	1.24 - 3.14
Comparison 2	-7.67	-8.27 - -7.07	-6.21	-7.0 - -5.45	1.81	0.86 - 2.76
Comparison 3	-11.62	-12.15 - -11.09	-14.48	-15.13 - -13.82	-3.10	-3.98 - -2.23
**Type of prescription**						
Western medicine	-	-	-	-	-	-
Chinese traditional medicine	7.11	6.72 - 7.49	7.22	6.77 - 7.67	15.56	14.61 -16.51
**Interaction - during**						
Other	-	-	-	-	-	
Intervention site * intervention period	2.78	2.13 - 3.43	8.84	8.00 - 9.68	-1.29	-2.29 - -0.30
**Interaction - post**						
Other	-		-	-	-	-
Intervention site * post-intervention period	8.89	8.27 - 9.52	18.59	17.77 - 19.40	-1.11	-2.07 - -0.15
**Gender**						
Male	-		-		-	-
Female	-3.12	-3.31 - -2.93	-4.75	-5.01 - -4.50	0.74	0.47 - 1.02
**Age (years)**						
< 5	-	-	-	-	-	-
5 < 15	5.95	5.54 - 6.36	7.28	6.71 - 7.85	3.21	2.69 - 3.72
15 < 25	11.90	11.46 - 12.34	15.61	15.05 - 16.17	9.25	8.56 - 9.93
25 < 35	14.51	14.10 - 14.92	18.60	18.08 - 19.12	10.85	10.18 - 11.52
35 < 45	15.63	15.23 - 16.04	19.58	19.06 - 20.11	10.30	9.70 - 10.90
45 < 55	17.13	16.71 - 17.56	21.06	20.51 - 21.61	10.72	10.11 - 11.33
55 < 65	18.56	18.11 - 19.00	22.36	21.76 - 22.95	11.81	11.20 - 12.41
65+	26.48	26.05 - 26.91	33.64	33.07 - 34.21	12.17	11.59 - 12.74
"adult"	13.84	13.29 - 14.40	9.58	8.11 - 11.05	7.87	7.31 - 8.43
unknown	6.12	5.31 - 6.93	11.38	9.49 - 13.28	2.42	1.64 - 3.20

Village clinic and THC outpatients only: multivariable regression analysis

## Discussion

Our hypothesis in undertaking this study was that any gender inequality in the use of health care would be reduced by the introduction of the CMS. Although we did observe that young girls and older women were relatively disadvantaged prior to the intervention, the effect of the intervention was equivocal. There was an increase in the female/male ratio for those under 5 years during the intervention but an unexpected deterioration for adult women. The strength of our conclusions must be tempered by the destruction of records, but as this was unlikely to have been biased by age or gender, it does not seriously undermine the validity of the results. We were, however, not able to test whether the absolute number of health care visits increased or decreased for either males or females (of any age), but we know from elsewhere that enrollment in a CMS tended to increase the overall number of patient visits and to increase (rather than decrease) out-of-pocket spending [5]. Given a limited pool of funds for health care within a family it is at least possible that increased out-of-pocket spending for one family member during the CMS would decrease the funds available for another, and that adult women would be relatively disadvantaged.

In reporting these results we are conscious that the number of health centre visits extracted represents only a small proportion of likely visits, particularly in the early years. From inquiries made during the fieldwork we believe this is largely due to the destruction or loss of registration books (by flood or fire, rodent infestation, sale for recycling or simply lost or thrown away), although it seems likely that some of the village clinics had, until recently, simply not kept registration records. Where books had been destroyed the loss would have affected males and females equally (once clinics for diseases specific to gender are excluded). A more serious flaw would arise if some clinic doctors had tended to register male patients more assiduously than female patients of the same age, and if this tendency had varied over time, being most pronounced in the intervention site during the intervention period: although possible, this does not seem a very credible alternative explanation for our findings. Our interpretation is also limited by a lack of information about the relative health care needs of men and women prior to the introduction of the CMS. Although our assumption was that women past childbearing would have poorer access to healthcare than other family members when all costs had to be met out-of-pocket, we have not attempted to demonstrate that there are unmet needs in this or indeed any group. It may be that the apparently greater use of services by men under the CMS is entirely a reflection of a poorer health status in men.

The study used a non-randomized design, in a controlled before and after study, with the use of comparison townsites to account for social and economic changes that occurred during the study period. Although it is recognized that such designs may be the only feasible option for evaluating some public health interventions [12,13] the evidence is weaker than would be obtained from a randomized design or even from a non-randomized design with multiple intervention sites: we do not know the full rationale for the choice of this county for the CNPAP (other than its poverty) or for the choice of the intervention township, although we can be fairly sure that it was not random. Although the use of comparison groups strengthens the design, there may be unmeasured confounders that provide an alternative explanation of the result observed and further testing of these findings would be valuable. Reports from other studies do not allow this directly, although there is some suggestion that insurance in rural China may affect differently the health or health care of men and women [14-16]. The absence of systematic studies of differential effects of the CMS on those of different age and gender is perhaps surprising, but computerized records arising from the new system may facilitate further inquiry.

The observed impact of a CMS on the prescription of certain types of drug and on prescription costs is in line with earlier reports. Sun et al found that prescription of antibiotics and use of intravenous medications was much greater where there was a CMS, with costs higher in these villages and higher also for members than non-members [8]. Wagstaff et al. compared data from the Chinese National Health Service Study for 10 counties with and 5 counties without CMS and found higher outpatient costs and greater out-of-pocket expenditures in those with CMS [5]. In the present study, although costs were regulated for drugs on the 'essential' list, alternative medications, such as newer antibiotics, could be substituted with the potential for greater profit. Our observation that the increase in costs was disproportionately in older males has not been reported previously.

It is important to consider how far results reported here may be generalized to the new CMS. First, it should be recognized that the short funding period for the China-Netherlands intervention may have lead to a pattern of health care use different from that in schemes planned to continue indefinitely. Second, reimbursement including outpatient costs is not typical of the majority of new CMS [6], which focus on inpatient care. Third although this part of Anhui is still poor, the non-health initiatives of the project may have served to reduce the level of poverty, a possible confounder as the impact of a CMS has been shown to differ by poverty level [5].

Much has been written about the need for change in regulation and for funding mechanisms that minimize health care decisions designed to augment the income of providers [5,14,17]. Such mechanisms are being put in place in rural China and indeed the county in which this study was based is now part of an experiment instituted by Anhui in which all drugs may only be charged at cost price. Any differential impact of the new CMS on older women would not be readily amenable to regulation while the family is still required to find the majority of health care costs out-of-pocket. Targeted incentives may be needed if real disparities in health care access are confirmed, with reimbursements greater for potentially disadvantaged subgroups.

## Conclusions

The study suggests that older women may get less access to health care under a cooperative medical scheme in which the major part of costs is met out-of-pocket. The study is observational and requires confirmation but underlines the need for future evaluative studies to use individuals, and not simply families, as the unit of analysis if such potential health disparities and to be identified and addressed. The effects of new funding on prescription practices and costs have now been found in a number of studies using different approaches. Interventions through regulation and/or new funding mechanisms, with planned evaluation of their impact, would seem well indicated.

## Competing interests

The authors declare that they have no competing interests.

## Authors' contributions

JQ JZ and NC contributed to the design, analysis, interpretation and writing of the report. ZM, TJ and LC contributed to the data collection, data preparation, data analysis and interpretation.

All authors read and approved the final manuscript.

## Pre-publication history

The pre-publication history for this paper can be accessed here:

http://www.biomedcentral.com/1471-2458/11/50/prepub

## Supplementary Material

Additional file 1 **Data available for analysis by period (before, during, after the intervention) and township**. Table showing the number of records extracted in each time period for each township Click here for file

Additional file 2 **Registrations by institution by period by township: gender determined disease and THC inpatients omitted**. Table showing the number of records extracted, and the proportion female, by institution, period and township. Click here for file
