# Supplementary material for: Evaluation of a pilot cooperative medical scheme in rural China: impact on gender patterns of health care utilization and prescription practices
Source: BMC Public Health. 2011 Jan 24;11:50. doi: 10.1186/1471-2458-11-50 (PMC3037865; doi:10.1186/1471-2458-11-50)
Supplement: Additional file 1 — Data available for analysis by period (before, during, after the intervention) and township. Table showing the number of records extracted in each time period for each township [file 1471-2458-11-50-S1.DOC]

|  | Intervention Township | | | Comparison Townships | | | | | | | | |
| --- | --- | --- | --- | --- | --- | --- | --- | --- | --- | --- | --- | --- |
|  |  | | | 1 | | | 2 | | | 3 | | |
|  | Registration | | Prescription | Registration | | Prescription | Registration | | Prescription | Registration | | Prescription |
|  | All | Reduced* |  | All | Reduced* |  | All | Reduced* |  | All | Reduced* |  |
| Period 1 (before)  Period 2 (during)  Period 3 (after)  Total | 5146  11769  19073  35988 | 4610  11095  17208  32913 | 23984  74178  83760  181922 | 30661  28943  25712  85316 | 28374  25838  21918  76130 | 3341  9444  35418  48203 | 19094  15173  16818  51085 | 16733  12907  14788  44428 | 8414  59017  103001  170432 | 3365  12767  13748  29880 | 2081  10491  11910  24482 | 99495  48146  91387  239028 |

*Reduced registration excludes those for diagnosis specific to gender (see text) and inpatients at township health centres.
