# Supplementary material for: Evaluation of a pilot cooperative medical scheme in rural China: impact on gender patterns of health care utilization and prescription practices
Source: BMC Public Health. 2011 Jan 24;11:50. doi: 10.1186/1471-2458-11-50 (PMC3037865; doi:10.1186/1471-2458-11-50)
Supplement: Additional file 2 — Registrations by institution by period by township: gender determined disease and THC inpatients omitted. Table showing the number of records extracted, and the proportion female, by institution, period and township. [file 1471-2458-11-50-S2.DOC]

| **Institution** | Intervention | | | Comparison | | | | | | | | | All Comparison | | |
| --- | --- | --- | --- | --- | --- | --- | --- | --- | --- | --- | --- | --- | --- | --- | --- |
|  |  |  |  | 1 | | | 2 | | | 3 | | |  |  |  |
|  | N | Female | % | N | Female | % | N | Female | % | N | Female | % | N | Female | % |
| **County hospital**  **Inpatient**  Period 1  Period 2  Period 3  **Outpatients**  Period 1  Period 2  Period 3  **THC**  Outpatients  Period 1  Period 2  Period 3  **Village Clinics**  Period 1  Period 2  Period 3  **All Institutions**  Period 1  Period 2  Period 3  **All Periods** | 175  316  300  120  114  276  1577  2031  3456  2733  8626  13144  4605  11087  17176  32868 | 62  117  100  60  63  132  733  950  1720  1338  4047  6154  2193  5177  8106  15476 | 35.4  37.0  33.3  50.0  55.3  47.8  46.5  46.8  49.8  49.0  46.9  46.8  47.6  46.7  47.2  47.1 | 140  294  179  124  97  168  25781  21475  18853  2325  3957  2706  28370  25823  21906  76099 | 40  100  50  62  44  64  11691  9929  8756  1128  2068  1352  12921  12141  10222  35284 | 28.6  34.0  27.9  50.0  45.4  38.1  45.3  46.2  46.4  48.5  52.3  50.0  45.5  47.0  46.7  46.4 | 83  136  125  89  112  130  15245  9866  12201  1313  2787  2320  16730  12901  14776  44407 | 28  42  45  38  44  70  7050  4831  5724  617  1385  1204  7733  6302  7043  21078 | 33.7  30.9  36.0  42.7  39.3  53.8  46.2  49.0  46.9  47.0  69.7  51.9  46.2  48.8  47.7  47.5 | 271  34.6  294  106  133  228  -  960  2146  1703  9038  9229  2080  10477  11897  24454 | 83  105  101  52  61  110  -  443  948  808  4441  4449  943  5050  5608  11601 | 30.6  30.3  34.4  49.1  45.9  48.2  -  46.1  44.2  47.4  49.1  48.2  45.3  48.2  47.1  47.4 | 494  776  598  319  342  526  41026  32301  33200  5341  15782  14255  47180  49201  48579  144960 | 151  247  196  149  244  545  18741  15203  15428  2553  7894  7005  21597  23493  22873  67963 | 30.6  31.8  32.8  47.6  43.6  46.4  45.7  47.1  46.5  47.8  50.0  49.1  45.8  47.7  47.1  46.9 |
